# Deciphering Auxin-Ethylene Crosstalk at a Systems Level

**DOI:** 10.3390/ijms19124060

**Published:** 2018-12-14

**Authors:** Elena V. Zemlyanskaya, Nadya A. Omelyanchuk, Elena V. Ubogoeva, Victoria V. Mironova

**Affiliations:** 1 Institute of Cytology and Genetics, Siberian Branch of the Russian Academy of Sciences (SB RAS), Novosibirsk 630090, Russia; nadya@bionet.nsc.ru (N.A.O.); ubogoeva@gmail.com (E.V.U.); 2 Department of Natural Sciences, Novosibirsk State University, Novosibirsk 630090, Russia

**Keywords:** phytohormone, transcriptional regulation, apical hook, root elongation, lateral root development, root hair formation, mathematical modeling

## Abstract

The auxin and ethylene pathways cooperatively regulate a variety of developmental processes in plants. Growth responses to ethylene are largely dependent on auxin, the key regulator of plant morphogenesis. Auxin, in turn, is capable of inducing ethylene biosynthesis and signaling, making the interaction of these hormones reciprocal. Recent studies discovered a number of molecular events underlying auxin-ethylene crosstalk. In this review, we summarize the results of fine-scale and large-scale experiments on the interactions between the auxin and ethylene pathways in Arabidopsis. We integrate knowledge on molecular crosstalk events, their tissue specificity, and associated phenotypic responses to decipher the crosstalk mechanisms at a systems level. We also discuss the prospects of applying systems biology approaches to study the mechanisms of crosstalk between plant hormones.

## 1. An Overview of Auxin and Ethylene Pathways in Plants

Auxin (indole-3-acetic acid, IAA) is a key regulator of plant development from cell growth and division to tissue specification and morphogenesis [1,2,3]. The essential role of auxin in governing developmental processes requires the establishment and maintenance of auxin gradients in tissues. This is achieved through the coordination of auxin biosynthesis, conjugation, and transport. In Arabidopsis, the *ANTHRANILATE SYNTHASE ALPHA SUBUNIT 1 (ASA1)* and *ANTHRANILATE SYNTHASE BETA SUBUNIT 1 (ASB1)* genes encode subunits of anthranilate synthase, which catalyzes the rate-limiting step in biosynthesis of *L*-tryptophan, the auxin precursor [4]. The key players in IAA biosynthesis are TRYPTOPHAN AMINOTRANSFERASE OF ARABIDOPSIS 1 (TAA1) and TAA1-RELATED (TAR) aminotransferases, which catalyze *L*-tryptophan deamination to form indole-3-pyruvate (IPA), and YUCCA (YUC) flavin-containing monooxidases, which promote IPA conversion to IAA. Auxin transport from the sites of auxin biosynthesis to the sites of its action is regulated by AUXIN-RESISTANT 1 (AUX1) and LIKE AUX1 (LAX) influx carriers [5,6], PIN-FORMED (PIN) efflux carriers (PIN1-4,7) [7], and a subset of ATP-binding cassette subfamily B (ABCB) transporters (ABCB1,4,14,15,19) [8]. In addition, PIN5,6,8 and PIN-LIKES (PILS) auxin transporters mediate intracellular auxin redistribution [9,10]. In the cell, auxin binding to nuclear TRANSPORT INHIBITOR RESPONSE 1 (TIR1) and AUXIN SIGNALING F-BOX (AFB) receptors promotes proteolytic cleavage of the AUXIN/INDOLE-3-ACETIC ACID (AUX/IAA) repressors, thereby depressing AUXIN RESPONSE FACTOR (ARF) family transcription factors, which trigger the transcriptional response to auxin.

Ethylene promotes numerous plant responses to changing environments, and various external signals induce its biosynthesis [11,12,13]. Ethylene is produced from *L*-methionine, which is consequently converted to *S*-adenosyl-*L*-methionine (by SAM-synthetases), 1-aminocyclopropane-1-carboxylic acid (ACC) (by ACC synthases) and ethylene (by ACC oxidases). It is noteworthy that ACC, being an ethylene precursor, might provide ethylene-independent regulatory effects as well [14]. Through the inactivation of the receptors (ETHYLENE RESPONSE 1 (ETR1), ETHYLENE RESPONSE SENSOR 1 (ERS1), ETR2, ERS2, ETHYLENE INSENSITIVE 4 (EIN4)) upon binding, ethylene blocks serine/threonine protein kinase CONSTITUTIVE TRIPLE RESPONSE 1 (CTR1) activity, thereby triggering the cleavage of EIN2 C-terminal domain (EIN2-C). EIN2-C then stabilizes EIN3 and EIN3-LIKE 1 (EIL1) transcription factors by translational repression of *EIN3 BINDING F-BOX1* and *2* (*EBF1* and *EBF2*) mRNA in cytosol [12], and facilitates EIN3 binding to the targets in the nucleus recruiting a histone binding protein EIN2 NUCLEAR ASSOCIATED PROTEIN 1 (ENAP1) [15,16]. Among other targets, EIN3 and EIL1 trigger the genes encoding various transcription factors, including multiple representatives of the APETALA2/ETHYLENE RESPONSE FACTOR (AP2/ERF) family, and thereby activate a transcriptional cascade [17].

## 2. Auxin-Ethylene Crosstalk at the Molecular Level

**Figure 1:**
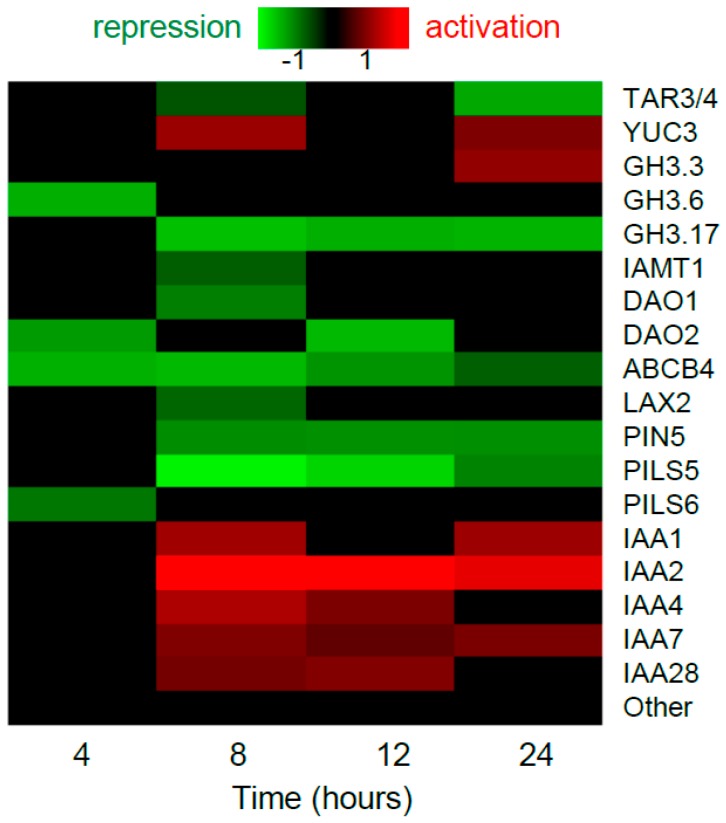
Transcriptional regulation of auxin-related genes by ethylene precursor ACC in Arabidopsis root. The datasets from Harkey et al. [20] were analyzed. Only genes with significant expression changes (Benjamini-Hochberg FDR < 0.05) are shown in the heatmap. We omitted the data for 0.5, 1 and 2 h time points, as there were only few differentially-expressed genes under this criterion. “Other” are the rest of genes from TAA1/TAR, YUC, PIN, Aux/LAX, PILS, TIR/AFB, ARF, Aux/IAA families and auxin-related genes from ABCB, GH3 families.

**Figure 2:**
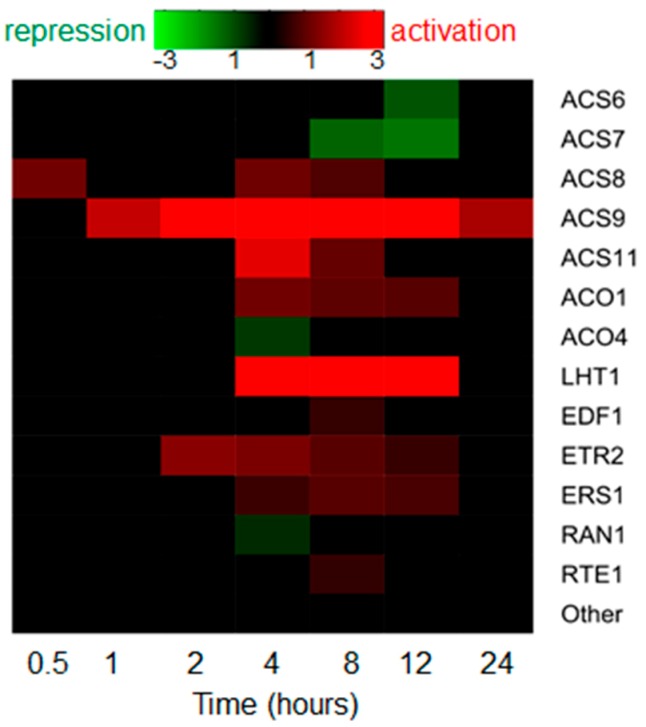
Transcriptional regulation of ethylene-related genes by IAA in the Arabidopsis root. The datasets from Lewis et al. [19] were analyzed. Only the genes with significant expression changes (Benjamini-Hochberg FDR < 0.05) are shown in the heatmap. “Other” are the rest of genes from ACS, ACO families and the genes involved in ethylene signaling and overviewed in chapter 1.

A coaction of auxin and ethylene is very important in fine-tuning various aspects of plant morphogenesis including root elongation, lateral root and root hair development, apical hook formation, and others [12,18]. In this section, we consider auxin homeostasis, transport, and signaling genes, which are the molecular targets for ethylene and vice versa, the ethylene pathway genes regulated by auxin. In addition to reviewing papers on this topic, we analyze publically-available datasets on the gene expression profiling of Arabidopsis roots treated with exogenous auxin IAA [19] or ethylene precursor ACC [20] over periods of 24 h. When analyzing these data, we consider the genes to be differentially expressed if they show significant changes in transcript levels with false discovery rate (FDR) controlled at 0.05 according to Benjamini-Hochberg procedure. Note that an absence of a gene in the list of differentially expressed genes (Figure 1 and Figure 2) does not guarantee that it is not regulated by the phytohormone at the transcriptional level; it might still be activated or repressed tissue- and condition-specifically.

### 2.1. Auxin Pathways Possess Ethylene Targets

Auxin pathways are widely involved in promoting ethylene responses. Accordingly, *Arabidopsis thaliana* mutants defective in auxin biosynthesis, transport, or signaling (e.g., *aux1*, *taa1/wei8*, *tir1*, etc.) demonstrate reduced sensitivity to ethylene treatment [12,18].

#### 2.1.1. Ethylene Regulates Expression of Auxin Homeostasis Genes

Among auxin biosynthesis genes, high ethylene levels cause an increase in an average amount of *ASA1* transcripts in roots [21], and *TAA1*, *TAR2*, *YUC3* and *YUC8* transcripts in seedlings [22,23]. An inhibitory effect of ethylene treatment was shown in seedlings for *YUC5* and *YUC6* genes [23]. Large-scale analysis of ACC effects on gene expression in Arabidopsis roots [20] showed upregulation of *YUC3* and downregulation of *TAR3/4* (Figure 1).

Auxin conjugation and oxidation serve to dampen cellular auxin level. No data has been published about the influence of ethylene on these processes; however, gene expression profiling of Arabidopsis roots [20] assumes that the influence occurs as ACC downregulated the genes encoding proteins catalyzing auxin conjugation (*GH3.6*, *GH3.17* and *IAMT1*) and oxidation (*AtDAO1* and *AtDAO2*), and it upregulated *GH3.3* (Figure 1).

Altogether, the large-scale data [20] suggest that ethylene has both positive and negative effects on auxin homeostasis. However, the underlying molecular mechanisms are largely unknown. The exception is ethylene-mediated upregulation of the *ASA1* gene, which is a direct target of EIN3-ERF1 transcriptional cascade [21].

#### 2.1.2. Ethylene Modulates the Abundance of Auxin Transporters

Polar auxin transport is the key factor to establish and maintain auxin gradients [7]. The genes encoding PIN efflux carriers (PIN1-4,7) and AUX1 influx carrier are upregulated in the roots upon ACC treatment [24,25]. Accumulation of *AUX1*, *PIN3*, and *PIN7* transcripts is *ETR1*- and *EIN2*-dependent [26]. Auxin upregulates these genes as well in a *TIR1*-, but not an *ETR1-* or *EIN2*-dependent manner. The fact that ACC induces *AUX1*, *PIN3*, and *PIN7* expression in *tir1* mutants evidences that ethylene and auxin regulate these genes through independent pathways [26]. The EIN3 binding region was detected in the promoter of *AUX1* gene, suggesting that it might be a direct target of the transcription factor [17]. *PIN2* was shown to be an indirect EIN3 target: it is upregulated by HB52 transcription factor, a direct target of EIN3 [27].

In the large-scale dataset introduced by Harkey et al. [20], only a small subset of auxin transporters was significantly downregulated by ACC at the transcriptional level. Intriguingly, three out of six ACC targets among auxin transporters (*PIN5*, *PILS5,6*) provide for intracellular auxin transport (Figure 1).

Ethylene regulates the abundance of auxin carriers at a posttranslational level as well. ACC treatment activates the genes encoding various kinases from AGCVIII family, including PINOID (PID), WAG1,2, and D6PKs (D6PK, D6PK1, D6PK2, and D6PK3) [27], the enzymes that phosphorylate PIN proteins and regulate their polarity and activity [28]. *WAG1* and *WAG2* are the other targets of the EIN3-HB52 transcriptional cascade [27]. These data suggest that a broad and poorly-analyzed network of ethylene-mediated trafficking of auxin carriers to the plasma membrane exists.

#### 2.1.3. Ethylene Affects Auxin Signaling

Mutations that cause defects in auxin signaling (*axr1*, *axr2/iaa7*, *axr3/iaa17*, and *tir1*) are resistant to ethylene inhibition of primary root growth, suggesting an essential role of auxin signaling in ethylene responses [24,25,29]. Auxin signaling not only implements ethylene effects through enhanced auxin biosynthesis and transport, but also represents a direct target of ethylene. Intriguingly, the large-scale dataset of Harkey et al. [20] demonstrated exclusively the activation effect of ACC treatment on *Aux/IAA* expression, while the expression of *ARF* genes was not affected (Figure 1). Moreover, *IAA2*, *IAA9*, and *IAA29* genes were detected as EIN3 candidate targets in a ChIP-seq experiment [17]. Additionally, Vaseva et al. [30] reported that the reduced ethylene signaling caused an impaired posttranslational response of Aux/IAA proteins to auxin in the root epidermis. All these facts suggest that ethylene might influence auxin signaling by modulating the level of Aux/IAA co-repressors in a cell. 

Several examples are known where EIN3 modulates auxin signaling via downstream effectors. EIN3 activates expression of *HOOKLESS1* (*HLS1*), a crucial regulator of apical hook development with a putative *N*-acetyltransferase activity [31,32]. In turn, HLS1 negatively regulates ARF2 levels, and may thereby change auxin-induced gene expression [33]. Another example is the regulatory chain EIN3-ERF72-ARF6, recently described as a part of BZR-ARF-PIF/DELLA-ERF (BAP/DE) module that controls hypocotyl growth at dark to light transition [34].

In a recent meta-analysis of auxin-responsive cis-elements over large-scale datasets, EIN3-binding core was detected as being specifically overrepresented in the promoter regions of the genes repressed by auxin in late response [35]. This data implies that ethylene might essentially contribute to the regulation of auxin downstream transcriptional cascade.

### 2.2. Auxin Reciprocally Regulates Ethylene Pathways

To date, either statistically insignificant [29] or only minor [24,30] disruptions of plant sensitivity to auxin were reported in mutants deficient in ethylene signaling. However, biochemical and molecular genetic studies demonstrate that auxin targets ethylene pathways as well.

#### 2.2.1. Auxin Drastically Affects Ethylene Biosynthesis

Auxin treatment enhances ethylene production in Arabidopsis seedlings [36,37]. Auxin upregulates the transcription of most of *ACS* genes, and alters their expression domains [37,38,39,40]. However, a systematic analysis of the large-scale datasets on IAA-induced Arabidopsis roots [19] suggests that the profile of IAA-dependent expression of ethylene biosynthesis genes is not so simple (Figure 2). Both positive and negative, early and late effects of auxin were shown for some *ACS* and *ACO* genes.

Rapid protein-synthesis-independent response of *ACS4* gene to auxin, and the presence of predicted ARF-binding sequences in the promoter regions of some *ASC* genes suggest that at least some of them might be direct targets of ARFs [29,38]. Besides, auxin differentially controls the turnover of ACS proteins: it stabilizes ACS2 and ASC5 enzymes, while no influence is detected for ASC7 [37].

Another player in auxin-ethylene crosstalk is REVERSAL OF SAV3 PHENOTYPE 1 (VAS1) aminotransferase that utilizes both IPA and *L*-methionine to produce *L*-tryptophan, and thereby suppresses both auxin and ethylene levels [41].

#### 2.2.2. Auxin Might Mediate ACC Transport

Being a gas, ethylene rapidly diffuses through the plant tissues to provide local responses. Ethylene long-distance transport occurs through aerenchyma. Alternatively, ACC is passively transported in vasculature [11]. A recent study [42] demonstrated that LYSINE HISTIDINE TRANSPORTER1 (LHT1) may promote ACC transport by facilitating ACC uptake under certain developmental or environment conditions [11]. In the large-scale dataset of Lewis et al. [19], *LHT1* is significantly upregulated by auxin (Figure 2).

#### 2.2.3. Auxin Modulates Ethylene Signaling

Auxin induces an increase in EIN3 stability [43]. This effect requires EBF1/2 function, but unlike ethylene, auxin does not alter EBF1/2 quantity to stabilize EIN3. However, primary roots of Arabidopsis mutants with partially or completely impaired ethylene signaling respond to auxin as wild-type (*etr1-3*, *ein2-5*, *ein3-1*, *eil1-1*) [24,29], or demonstrate only slightly reduced root sensitivity to auxin treatment (*ein2-1*) [24,30]. Thus, unlike ethylene, which actively involves auxin to achieve its morphogenetic effects, auxin scarcely requires ethylene signaling to perform its functions. A moderate effect of auxin on ethylene signaling is supported by the data from Lewis et al. [19], since only a minor fraction of genes from ethylene signaling responded to auxin, and only mild changes were detected (Figure 2).

## 3. Auxin-Ethylene Crosstalk at the Systems Level

In this section, we consider the tissue context for the molecular events guided by auxin-ethylene crosstalk, and review the correlations between them and phenotypic responses. Additionally, we make an attempt to classify the mechanisms of phenotype formation due to auxin-ethylene crosstalk.

### 3.1. Ethylene-Induced Auxin Accumulation: Inhibition of Root Elongation

Morphogenetic effects of ethylene can be promoted through local accumulation of auxin in a plant tissue upon activation of auxin biosynthesis and transport [24,25]. A typical example is the suppression of Arabidopsis root elongation after application of ethylene or its precursor ACC.

**Figure 3:**
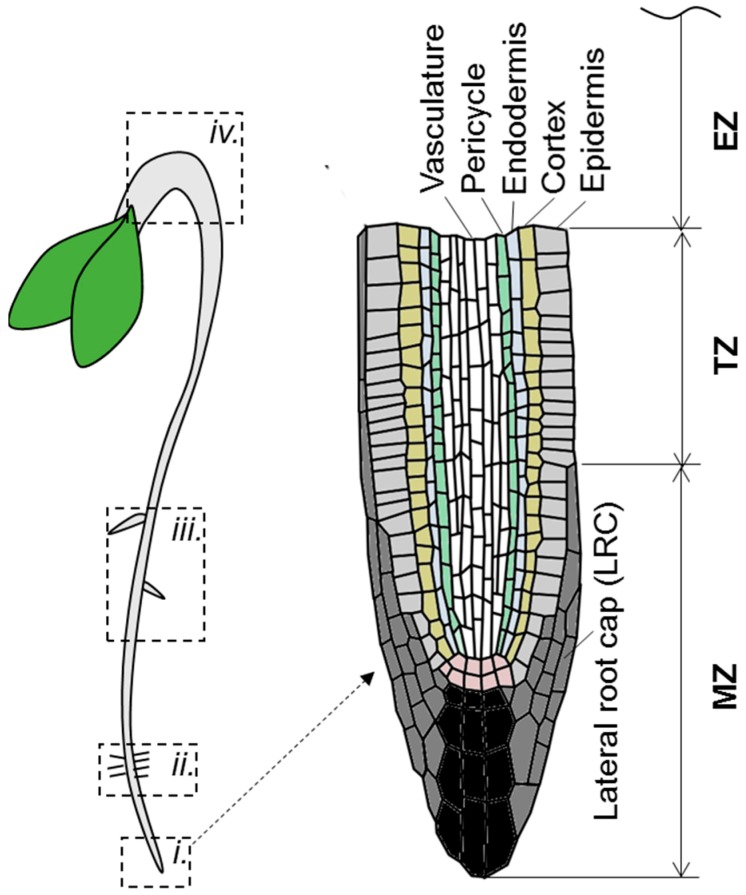
Schematic representation of Arabidopsis seedling structure. On the left panel the root top (***i.***), root hair forming region (***ii.***), the zone of mature root (***iii.***) and apical hook (***iv.***) are highlighted. On the right panel the root tip structure is detailed. MZ—meristematic zone; TZ—transition zone; EZ—elongation zone.

In the root tip, ACC enhances the signal of auxin sensors (e.g., *DR5*- and *pIAA2*-driven reporters), and causes their ectopic expression in the lateral root cap and epidermis of the root meristematic and elongation zones (Figure 3), implying local auxin accumulation in these tissues [24,25]. These changes strongly correlate with ethylene-dependent inhibition of root elongation. For example, *ctr1* mutants having constitutive ethylene response exhibit the short root phenotype and the permanent ectopic expression of *pIAA2:GUS* at the root apex [25]. Conversely, in ethylene-insensitive plants (e.g., in wild type seedlings treated with silver ions, *etr1-3*, *ein2*, and *aux1* mutants), ethylene fails to induce any changes [24,25,44].

**Figure 4:**
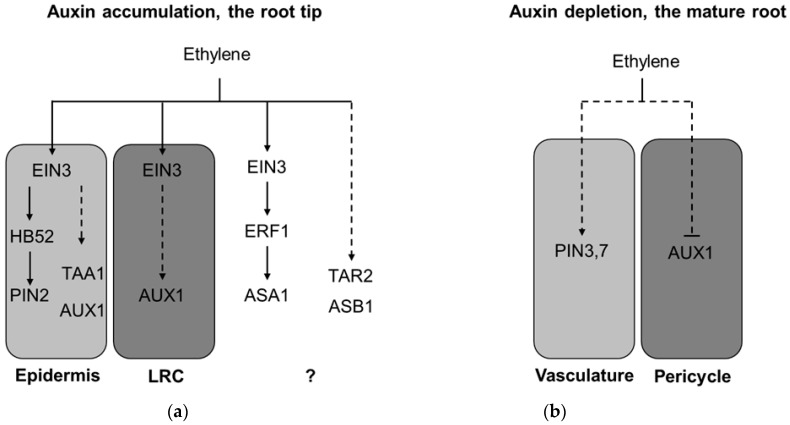
The models of ethylene-induced auxin accumulation in the root tip (**a**) and ethylene induced auxin depletion in the root mature zone (**b**). (**a**) To regulate root growth, ethylene principally targets LRC and epidermis in the meristematic and early elongation zones in the root tip (zone *i.* in Figure 3). Upon perception in these tissues, ethylene affects local auxin biosynthesis through TAA1 accumulation in epidermal cells and *ASA1*, *ASB1*, and *TAR2* induction (tissue specificity is unknown), and enhances shootward auxin transport in the LRC and epidermis by increasing AUX1 and PIN2 abundance. As a result, auxin accumulation in the outer layers of the root tip restricts root elongation. This model is based on the findings reported previously [21,22,24,25,27,30,44]. (**b**) In parallel with (**a**) ethylene induces PIN3 and PIN7 accumulation in the central cylinder of the whole root and blocks local AUX1 accumulation in the pericycle cells at the sites of lateral root initiation in the mature root (zone *iii.* in Figure 3). As a result, both enhanced rootward auxin transport through the central cylinder and reduced local auxin uptake in the pericycle prevent local auxin maxima formation required for lateral root primordia initiation. This model is based on the findings reported in Lewis et al. [26]. Dashed lines mark regulatory events with unknown mechanisms. LRC—lateral root cap. Question mark denotes the regulations with unknown tissue specificity.

Ethylene regulates root elongation by fine-tuning auxin biosynthesis and transport in a tissue-specific manner (Figure 3 and Figure 4A). The ethylene signal is perceived in the lateral root cap (LRC) and epidermal cells in meristematic, transition, and early elongation zones [30]. Upon perception, ethylene promotes local auxin biosynthesis by increasing TAA1 abundance in expanding epidermal cells [30]. Induction of *ASA1*, *ASB1*, and *TAR2* auxin biosynthesis genes in response to ethylene was also reported in the root tip [22,44]; however, it remains to be investigated whether it is tissue-specific or not.

At the same time, ethylene enhances shootward auxin transport by inducing accumulation of AUX1 and PIN2 in the outer layers of the root tip [24,26,30]. Elevated expression of AUX1 in LRC cells rather than in epidermis is crucial for the inhibition of root growth by ethylene [25]. Such fine-tuning of auxin biosynthesis and transport from the root apex via the lateral root cap to elongating epidermal cells results in an apoplastic alkalinization, which inhibits cell elongation [30,45]. Additionally, exogenous ACC triggers the expression of ethylene sensor *EBS:GUS* in the root elongation zone in *AUX1*-dependent manner suggesting the possible role of reciprocal regulation in root responses to ethylene [29]. The roles of auxin transport and local auxin production in regulation of root growth are complementary though distinct, because shootward auxin transport cannot fully compensate auxin production in epidermis [46]. The model also proposes that ethylene-dependent epidermis growth subsequently drives the growth of inner root tissues [30].

### 3.2. Ethylene-Induced Auxin Depletion: Inhibition of Lateral Root Initiation

Besides auxin accumulation in the root tip, ethylene induces auxin depletion in the mature regions of root, thus blocking lateral root initiation (Figure 3 and Figure 4B) [26,47]. Upon ACC treatment, PIN3 and PIN7 auxin transporters are upregulated in the root mature zone, leading to elevated rootward auxin transport that prevents formation of *DR5* expression maxima in pericycle cells important for lateral root initiation. In addition, ACC blocks AUX1 expression in the mature root regions and at the root bends [26], which normally concentrate auxin maxima in the forming lateral root primordia [48], that serves to suppress auxin uptake in ethylene-dependent inhibition of lateral root initiation. Thereafter, the sensitivity of lateral root development to ACC treatment is suppressed in *aux1*, *lax3*, *pin3*, and *pin7* mutants [26,47].

Nevertheless, the treatment of seedlings with low ACC doses has an opposite effect compared to high ACC doses, and promotes the initiation of lateral root primordia [49]. It was hypothesized that, during root development, low ethylene concentrations produced in differentiating protoxylem vessels might trigger lateral root initiation in auxin-dependent manner [50]. However, the molecular mechanisms of such activation remain elusive.

### 3.3. Ethylene-Mediated Asymmetry in Auxin Distribution: Apical Hook Formation

**Figure 5:**
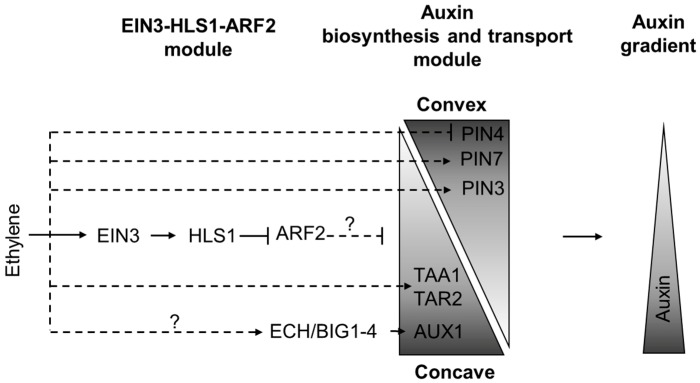
The model of ethylene influence on auxin distribution asymmetry during apical hook formation. In the hook (zone *iv.* in Figure 3), ethylene activates expression of *HLS1*, which negatively regulates ARF2 levels to mediate asymmetric auxin distribution. Ethylene treatment downregulates PIN4, upregulates PIN3 and PIN7 expression in the epidermal cells of the hook. Simultaneously *TAR2* and *AUX1* expression is induced on the concave side of the apical hook. As a result, ethylene fine-tunes auxin maximum formation on the concave side of the hook. Dashed lines mark the regulatory events with unknown mechanisms, question marks highlight putative regulations. The triangles conditionally depict spatial molecular gradients. This model is based on findings reported previously [12,53,54].

The morphogenetic effects of ethylene can also be caused by asymmetry in auxin distribution [12,51]. The principles of ethylene action in these cases are similar to those described above—upregulation of auxin biosynthesis and transport; however, the sites of auxin accumulation locate in tissue asymmetrically. For example, in dark-grown Arabidopsis seedlings, ethylene mediates asymmetric auxin distribution in the apical hook, and thereby contributes to the hook formation (Figure 3 and Figure 5) [51,52].

PIN3 supplies auxin from the central cylinder to outer hypocotyl tissues, while asymmetric *PIN4* and *PIN7* expression on the convex side of the hook in both the cortex and epidermis promotes auxin transport toward the concave side of the hypocotyl that is sufficient to generate the auxin maximum in the epidermal cells at this side [53]. When exogenously applied, ethylene reduces *PIN4* expression, elevates *PIN7* signal on both sides, and slightly enhances *PIN3* asymmetry between the convex and concave sides of the hook. Ethylene also induces *TAR2* and *AUX1* expression on the concave side of the hook [22,55]. All these changes mediate auxin maximum establishment on the concave side of the hook [53,55]. As a result, exogenous treatment of dark-grown seedlings with ACC prolongs the formation phase of the hook development and exaggerates apical hook curvature [12,56]. Accordingly, *aux1*, *pin3* and *wei8/taa1 tar2* mutants do not demonstrate exaggeration of apical hook in response to ethylene [21,55,56].

Ethylene stimulates AUX1 turnover in plasma membrane specifically on the concave side of the apical hook [55]. ECHIDNA (ECH) and BIG1-4 proteins mediate AUX1 vesicle trafficking and, in this way, are also involved in auxin-ethylene crosstalk during apical hook development [57]. Respectively, *ech* and *big1-4* mutants show ethylene-resistance coupled with impaired AUX1 expression [54,58].

Another player which contributes to auxin-ethylene crosstalk during apical hook development is *HLS1*, a direct EIN3 target [31,32]. Mutants with strong *hls1* alleles stay hookless upon ethylene treatment [31,32]. To positively control hook formation, HLS1 negatively regulates ARF2 levels [33], and influences auxin distribution [31]. The exact mechanisms that connect HLS1 to auxin distribution are still unknown.

### 3.4. The Convergence of Auxin and Ethylene Pathways in Root Hair Development

The root transcriptome profiling in response to auxin and ethylene showed that half of the targets common to both hormones are triggered by ethylene independently of auxin, and 30% are triggered by auxin in an ethylene-dependent manner [29]. This supports the idea that at least part of ethylene effect in the root should be auxin-independent. The development of root hairs is one of the examples demonstrating this idea. Elevated levels of auxin or ethylene intensified root hair initiation and elongation [12,18]. However, elevated levels of ethylene but not auxin induce ectopic root hair cells, implying a specific role of ethylene in root hair morphogenesis [59].

**Figure 6:**
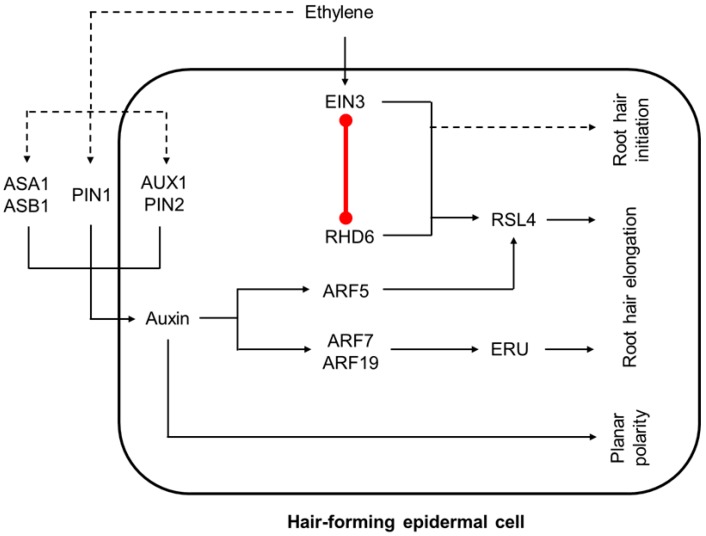
The model of auxin-ethylene crosstalk during root hair development. Auxin redistribution contributes to the ethylene effects on planar polarity of root hair-forming cell and root hair growth (zone *ii.* in Figure 3). However, ethylene acts independently of auxin redistribution as well. EIN3 directly interacts with RHD6 to regulate root hair initiation and elongation. Dashed lines mark the regulatory events with unknown mechanisms. Red line marks protein-protein interaction. This model is based on findings reported previously [59,63,64,65].

Root hair development includes the cell fate choice, root hair initiation, and its elongation via tip growth [60]. The fates of epidermal hair-forming and non-hair-forming cells are determined by a position-dependent signaling [60,61]. After specification, establishment of planar polarity within a cell permits selection of the root hair initiation site at the more basal (towards the root tip) part of the cell, and auxin gradient plays a crucial role in this process [61]. Next, auxin controls root hair tip growth by directly inducing two key players: root hair elongation regulator *ROOT HAIR DEFECTIVE 6-LIKE 4* (*RSL4*) via ARF5 [62], and root-hair-specific cell wall regulator ERULUS (ERU) kinase via ARF7 and ARF19 [63] (Figure 3 and Figure 6).

On one hand, ethylene regulates planar polarity of root hair cell and root hair tip growth upstream of auxin by activation of auxin biosynthesis (*ASA1*, *ASB1*) and transport (*AUX1*, *PIN2*, *PIN1*) genes as discussed above [64,66]. On the other, the role of ethylene in regulation of root hair development is not restricted to auxin redistribution (Figure 6). Particularly, *arf7 arf19* double mutant defective in both auxin signaling and root hair development can be rescued by ACC treatment [67].

Auxin and ethylene pathways converge to activate the *RSL4* gene—a direct ARF5 target, which controls root hair elongation. EIN3 physically interacts with ROOT HAIR DEFECTIVE6 (RHD6), a major regulator of root hair initiation, and it directly coactivates *RSL4* [65]. Accordingly, auxin and ethylene have very similar transcriptional responses in root epidermis, and the majority of their common target genes are positively regulated by RSL4 [59,68,69]. At the same time, the role of EIN3-RHD6 cooperative action is most likely not limited to *RSL4* regulation, but also contributes to ethylene-promoted root hair initiation [65]. Moreover, EIN3-RHD6 cooperation might be important for auxin functioning in root hair initiation, as exogenous auxin application fails to rescue hairless phenotype of *ein3 eil1 rhd6 rsl1* quadruple mutants [65].

## 4. Mathematical Modeling of Auxin-Ethylene Crosstalk

The observations described above highlight the complex nature of auxin-ethylene crosstalk. Computer modeling is a powerful tool for tackling the complex regulatory mechanisms that make it possible to support or reject the hypotheses proposed based on experimental observations, and to make predictions to design novel experiments.

Auxin-ethylene crosstalk in the cell has been studied with experimental and modeling approaches in [70]. In the single-cell model, positive feedback between auxin and ethylene biosynthesis were attenuated with mutually inhibitory auxin-cytokinin interactions and with the positive regulation of ethylene by cytokinin. The model studied the role of POLARIS (PLS) peptide in hormonal crosstalk circuit. *PLS* gene expression is activated by auxin signaling, and the peptide restricts several ethylene-mediated processes, including growth in the dark, polar auxin transport, auxin homeostasis, and microtubule dynamics [71,72]. The model predicted that PLS protein modulated not the ethylene level, but ethylene signaling. It was also shown that PLS controls the way in which ethylene regulates cellular auxin concentration affecting either auxin transport or auxin biosynthesis.

Simplified auxin-ethylene-cytokinin crosstalk [70] was studied with a spatiotemporal resolution in two-dimensional multicellular root structure [73]. The authors additionally considered ethylene- and cytokinin-regulated AUX1 expression. The model reproduced auxin patterning in the root tip of wild type and ethylene-sensitive mutants. It also predicted the amounts of cytokinin, ethylene, AUX1 and PIN proteins, which were successfully verified in the experiment. Recently the same authors discussed the complexity of the crosstalk network, demonstrating that only small part of the network was integrated in the mathematical model and analyzed in dynamics [74].

Another successful example of auxin-ethylene crosstalk modeling is the simulation of asymmetric auxin distribution during apical hook formation [53]. Both in vivo and in silico studies showed that asymmetric expression of the PIN auxin transporters at the concave versus convex side of the apical hook is sufficient for establishing an auxin maximum in the epidermis at the concave side. Meantime reproducing in silico exaggerated hook curvature formed after exogenous ethylene treatment was possible only after enlargement of cell proliferation zone in the model [53], i.e., the phenotype previously reported by Raz and Koornneef [75]. Increase in expression of cell division regulatory genes in the apical hook after ethylene treatment also confirmed the model prediction [53]. Nevertheless, a dramatic reduction of cell proliferation zone was observed in *shy2/iaa3*, *slr/iaa14* and *pin3* mutants, and these defects could not be fully rescued by ethylene treatment, indicating that auxin signaling was also required to maintain cell proliferation. Thus, the combination of experimental and modeling approaches indicated that during apical hook development, auxin and ethylene jointly coordinate differential cell division and elongation.

## 5. Conclusions

Auxin and ethylene cooperatively regulate many developmental processes in plants. To date, a lot of information is available on the molecular events promoting auxin-ethylene crosstalk at the levels of biosynthesis, transport, and signaling. This includes transcriptome profiling datasets of which meta-analysis makes it possible to gain an overview of the genes responding to auxin and ethylene, and to determine new candidates for the molecular crosstalk. By affecting auxin biosynthesis and transport, ethylene promotes auxin accumulation, depletion, or asymmetric redistribution in plant tissues, and thereby, triggers morphogenetic responses. However, the role of ethylene in the crosstalk is not restricted to auxin redistribution, and one of the challenges is to unveil new types of auxin-ethylene interactions.

The other point for future research is the role of auxin signaling in ethylene responses. On one hand, assuming that a certain ethylene-induced phenotype is a consequence of altered auxin distribution, it would be logical to expect that ethylene regulates it through downstream auxin signaling. However, distinct auxin signaling elements may be required for different ethylene-induced phenotypic changes. Moreover, auxin signaling elements, recruited by ethylene and auxin to regulate similar phenotypes, may differ. Additionally, auxin signaling components might be specifically triggered by ethylene, as in the case of the EIN3-HLS1-ARF2 regulatory chain (Figure 5).

Another gap is the role of reciprocity of auxin-ethylene crosstalk; the physiological role of this influence is still obscure. Most likely, one or more factors exist that suppress auxin-induced ethylene responses. This is in a good accordance with the overall trend of preventing ethylene response from getting out of control upon the activation of positive feedback loops, because the uncontrolled activation of ethylene signaling might cause severe developmental perturbations, including premature senescence and tissue death. Additional regulatory modules for this tight control of auxin-induced ethylene signaling might be sought.

Mathematical modeling is a powerful approach to explore the complex regulatory mechanisms at a systems level.
